# Experienced and internalized weight stigma among Spanish adolescents

**DOI:** 10.1186/s12889-024-19246-7

**Published:** 2024-06-29

**Authors:** Dimitra Anastasiadou, Salomé Tárrega, Albert Fornieles-Deu, Albert Moncada-Ribera, Anna Bach-Faig, David Sánchez-Carracedo

**Affiliations:** 1 Eating and Weight-Related Problems Unit, Universitat Autònoma de Barcelona, Edifici B. Campus de la UAB. 08193, Bellaterra (Cerdanyola del Vallés), Barcelona, Spain; 2 Department of Clinical and Health Psychology, Universitat Autònoma de Barcelona, Edifici B. Campus de la UAB. 08193, Bellaterra (Cerdanyola del Vallés), Barcelona, Spain; 3 Department of Epidemiology and Methodology of Social and Health Sciences, Faculty of Health Sciences at Manresa, Universitat de Vic – Universitat Central de Catalunya (UVic- UCC), Av. Universitària, 4-6, Manresa, 08242 Spain; 4 Research Group in Epidemiology and Public Health in the Digital Health context (Epi4Health), Institute for Research and Innovation in Life and Health Sciences in Central Catalonia (IRIS-CC), Ctra. De Roda Núm. 70, Vic, 08500 Spain; 5 Department of Psychobiology and Methodology of Health Sciences, Universitat Autònoma de Barcelona Serra Húnter fellow, Edifici B. Campus de la UAB, 08193, Bellaterra (Cerdanyola del Vallés), Barcelona, Spain; 6 Head of Community and Health Service, Social Rights Section, City Council of Terrassa, Ca. Torres Garcia 35, Terrassa, Barcelona 08221 Spain; 7 Food Lab Research Group, Faculty of Health Sciences (2021 SGR 01357), Open University of Catalonia (UOC), Barcelona, 08018 Spain

**Keywords:** Weight stigma, Wight bias internalization, Adolescents, Weight status

## Abstract

**Background and objective:**

Weight stigma has negative consequences for both physiological and psychological health. Studies on weight stigma in adolescence, particularly from general populations, are scarce in the Mediterranean area. The main aim of this study is to describe the prevalence of experienced and internalized weight stigma among a representative sample of adolescents from the Spanish city of Terrassa, and to determine its association with sociodemographic variables and weight status.

**Methods:**

Drawing on data from the initial assessment of a longitudinally funded project on weight stigma in adolescents, a cross-sectional survey-based study was conducted using random multistage cluster sampling. Weight stigma experiences, their frequency and sources, and weight bias internalization with the Modified Weight Bias Internalization Scale (WBISM) were assessed in a sample of 1016 adolescents. Adjusted odds ratios (AOR) between sociodemographic variables, weight status and having experienced weight stigma, and having reported high scores of WBISM (WBISM ≥ 4) were estimated by multiple logistic regression models.

**Results:**

The prevalence of weight-related stigma experiences was 43.2% in the sample (81.8 in adolescents with obesity) and the prevalence of high levels of weight bias internalization was 19.4% (50.7 in adolescents with obesity). Other kids and school were the most prevalent sources of weight stigma, with society and family being other significant sources of stigma reported by girls. A significantly higher risk of having experienced weight stigma was observed in girls (AOR = 2.6) and in older adolescents (AOR = 1.9). Compared to normal weight adolescents, all weight statuses showed higher risk, being 3.4 times higher in adolescents with underweight and reaching 11.4 times higher risk in those with obesity. Regarding high levels of weight bias internalization, girls had a risk 6.6 times higher than boys. Once again, a “J-shaped” pattern was observed, with a higher risk at the lowest and highest weight statuses. The risk was 6.3 times higher in adolescents with underweight, and 13.1 times higher in adolescents with obesity compared to those with normal weight.

**Conclusions:**

Considering the high prevalence of experienced and internalized weight stigma among adolescents in Spain, especially in adolescents with obesity and girls, it seems important to implement preventive strategies in different settings and address all sources of stigma.

## Introduction

Obesity is one of the greatest public health concerns of the 21st century [1] that compromises health in its broader spectrum, including physical [2] and mental well-being [3]. Regarding its prevalence, several countries worldwide have experienced a double or triple escalation of obesity among the adult population in the last three decades while an alarming increase has also been observed at younger ages [4]. More precisely, in Spain, it is estimated that more than 30% of people aged 3 to 24 years are living with overweight or obesity [5].

Preventive and treatment strategies primarily follow a “weight normative approach” [6], attributing the origin and controllability of the problem to the proper individual. This approach, which prioritizes lifestyle-based weight-centric paradigms, is questionable [7] and is often associated with binge eating and unhealthy weight control behaviors, poor mental health [8], and body dissatisfaction [9] This is particularly noteworthy during adolescence, considering the importance of identity construction occurring in this period and its complex interaction with the internalization of the thin ideal and body dissatisfaction [10]. In contrast, the “weight-inclusive approach,” emphasizing a multifaceted view of health and well-being while striving to enhance health access and reduce weight stigma, appears more advantageous. This is particularly evident given the high rates of weight regain and cycling associated with weight loss interventions under the “weight-normative approach” [7].

Under this weight normative approach, weight stigma is cultivated, expands across multiple facets of everyday life [11], and can lead to negative attitudes, stereotypes, and discrimination, with children and adolescents being one of the most vulnerable groups for weight stigmatization [12]. For instance, stereotypes related to the fact that people living with obesity are viewed as lazy, lacking motivation to change or self-discipline, are highly tolerated given that it is wrongly assumed in modern societies that stigma and shame would motivate people towards a healthier lifestyle change [13, 14]. All in all, weight stigma persists despite the alarmingly growing prevalence of obesity nowadays and is considered an additional psychosocial contributor to the problem [12].

Weight stigma experiences among adolescents are present in different settings, including school, home, healthcare, and mass media [13, 15]. Evidence shows that these experiences (including weight-based discrimination, teasing, or bullying -verbal or physical-), rather than encouraging healthy behavioral change, are associated with social isolation, body dissatisfaction, and mental health problems, among other long-lasting harmful consequences [13, 16]. At the same time, weight bias internalization (WBI), referring to the process of being aware and agreeing with negative weight-based stereotypes, applying these to oneself and engaging in self-blame and self-devaluation for weight [17], may create an additional challenge to the effective management of obesity given that it is associated with worse psychosocial, physical, and behavioral health [18, 19] and has been associated with maladaptive eating behaviors and poor psychological health in youth [20].

To date, research on weight stigma has been mainly focused on adults and samples of people living with obesity engaged in weight loss programs. For instance, research conducted with adults from the general population reported a high prevalence of weight stigma experiences (above 40%) and high levels of WBI (around 20%) [21, 22], with a higher risk of both types of stigmas among women, those who were categorized as overweight or with obesity, and those who believed that individuals were personally responsible for their body weight [21]. However, studies on weight stigma in adolescence, particularly from general populations, are still scarce [23] while in Spain, and in the Mediterranean area in general, research in this field is notably limited. Furthermore, the few prevalence studies in this field have been conducted with convenience community samples, both with adults and adolescent samples [21, 22, 24–26]. To our knowledge, only one study has been conducted with adolescents in Spain, showing the negative impact of WBI on adolescents´ self-esteem and body satisfaction across all genders and weight categories, with the exception of the underweight group [27].

To develop weight stigma-reduction initiatives at young ages, it becomes increasingly important to shed more light on how weight stigma experiences and WBI are associated with weight status, health outcomes, and emotional well-being in adolescents and to better explore key contextual factors involved in this association [16]. The aim of the present study is to describe the prevalence of experienced and internalized weight stigma among a representative sample of adolescents (11–16 years) from the Spanish city of Terrassa, and to determine its association with sociodemographic variables and weight status. As we do not have prevalence data on this issue in other Mediterranean samples, it is not easy to establish hypotheses. However, based on previous studies with other types of populations, regarding sociodemographic determinants, our exploratory hypothesis is to find a higher frequency of experienced stigma and higher levels of internalized stigma in the female sample and in adolescents with obesity.

## Materials and methods

### Design and participants

**Fig. 1:**
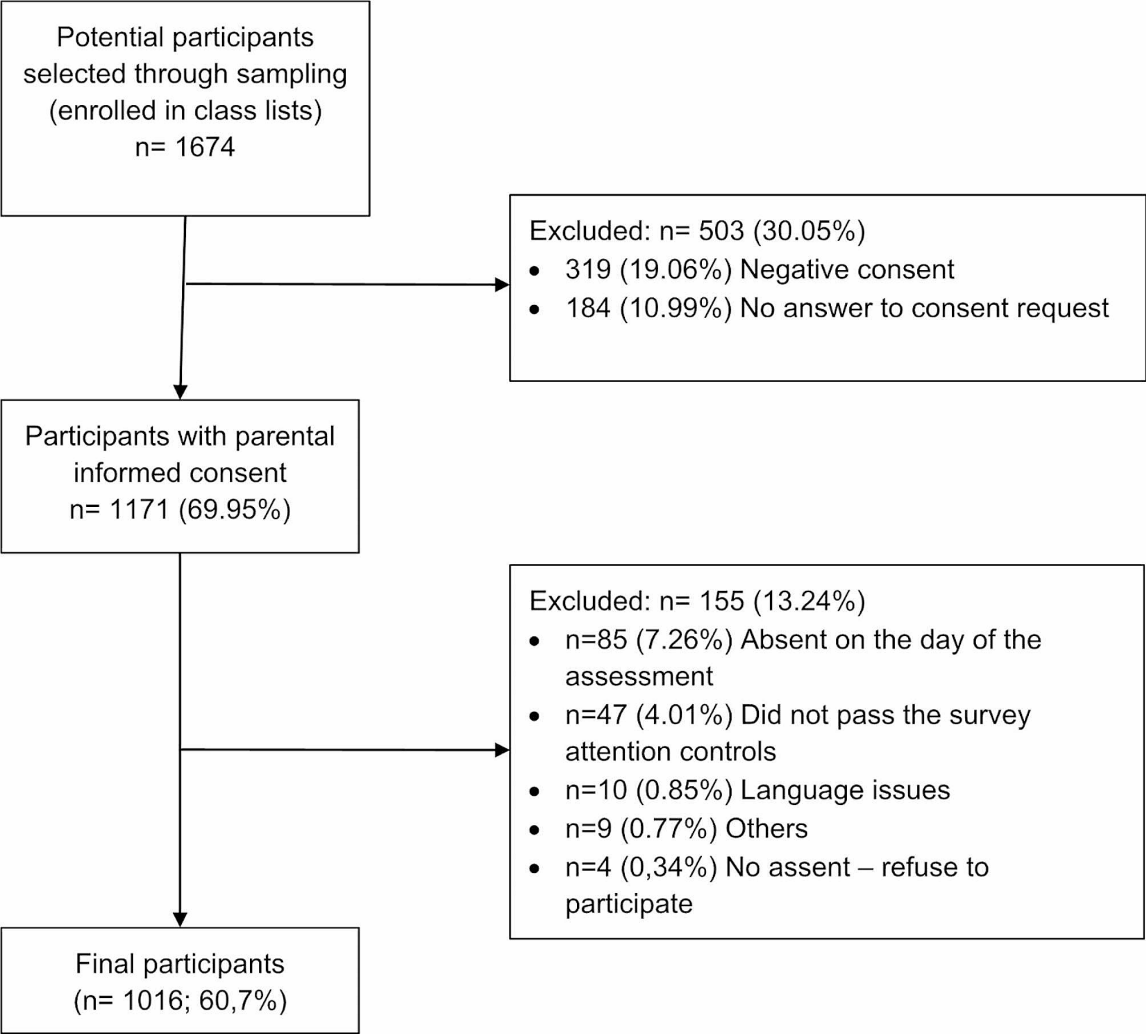
Participants’ flow diagram

This is a cross-sectional survey-based study. This study is based on data from the first assessment of a longitudinal funded project on weight stigma in adolescents. The final sample of this study consisted of 1016 adolescents enrolled in secondary schools from Terrassa, the third most populous city in Catalonia, area of Barcelona, Spain. The adolescents in our study were from the four years of Compulsory Secondary Education in the Spanish system (11–16 years). The subsequent educational stage (16–18 years) is not compulsory in Spain. As our intention was to select a representative sample, we decided to include only adolescents who were enrolled in mandatory education, since all adolescents are in school at this stage. Schools (7 public schools and 9 grant-aided schools) and one classroom for each course (total of 64 classrooms) were selected using a random multistage cluster sampling to obtain a representative sample of the city. The sample size estimation for our study was performed with the objective of reaching a final sampling error of around 3%. Given a total population of 10,097 students enrolled in Compulsory Secondary Education (ESO) in the academic course 2021-22, and assuming a maximum uncertainty with a confidence level of 95.5% (p = q = 0.5 and 2σ), we initially calculated a sample size of approximately 1,400 participants to achieve a sampling error of 2.48%, lower than expected to allow for the possible loss of participants. Our final sample of 1,016 subjects resulted in the expected sampling error of 3%. No specific exclusion criteria were used, and all adolescents who were present at the time of the assessment and had parental informed consent participated. Only participants who did not have parental informed consent, refused to participate, did not respond to the parental informed consent request, or were invalid (because of language issues or because did not pass the survey controls) were excluded from the original class lists. Figure 1 shows the flow diagram of the sample.

### Procedure

The study was supported by the Community and Health Service of the City Council of Terrassa, with which the sampling of the participating schools was made, and which mediated to obtain the participation of the schools. The Principal Investigator of the project held meetings with the management teams of selected schools to present the project, establish the assessment calendars and obtain the lists of the enrolled students of the selected classrooms, with their initials to maintain confidentiality. The legal guardians’ informed consent and participants’ assent were obtained. A code was assigned to each potential participant and noted on the informed parental consent sheets. Between January and February 2022, the tutors of each selected classroom gave these sheets to each participant, along with a sheet with information about the study. The participants took it home and were given a period of one month to return the informed consent sheets with the families’ responses. An active response was requested (Yes/No). The assessments were carried out in April and May 2022. The design of the survey format was carried out on a specific online platform of the company Digital Insights S.L., with forced-response, and incorporating controls for response ranges and two interspersed control questions to verify the level of attention of the participants (by requesting them to answer one option in the question). The missing data were avoided with this system. A group of technicians, specifically hired for the project and previously trained (graduate psychologists), supervised the administration of the online survey in the classrooms. First, they entered in duplicate the code assigned to each participant with positive parental informed consent. For adolescents without positive parental informed consent, they were relocated to another classroom under the supervision of a teacher. After informing the participants about the study, their voluntary participation, and their right to withdraw at any time without providing reasons and without negative consequences, they were asked to respond to the request for informed consent (Yes/No) in the first question of the survey, The survey was made up of a wide inventory of questions and questionnaires validated to assess variables of interest to the project. Another group of technicians gathered groups of 5–7 adolescents from the classroom, moving them to a private area, where measurements of height and weight in light clothes and barefoot were taken following a standardized protocol [28] and recommendations by the Catalan Public Health Agency [29]. After the height and weight measurements were taken, the participants returned to the classroom to complete the survey. When finished, the technicians entered the weight and height measurements of each participant and a code to end the survey. To maintain confidentiality, participants could only access this information at that time upon request, thereby avoiding comments about weight and height. The assessment sessions lasted one hour per classroom. Data was pseudo-anonymised. The confidentiality of the participants was protected with numeric codes and data processing was conducted anonymously, but the principal investigator, the only one with access to the codes and class lists, can identify the cases to assign them the same code in a future second measurement, forming part of a longitudinal study. The participating schools received a detailed report of the main descriptive results at the beginning of the following academic year, fulfilling a commitment made to them. The data belongs to a larger database that has not yet been fully exploited, however, the data set is available upon request to authors. This study was carried out in accordance with the guidelines established in the Declaration of Helsinki of the World Medical Assembly [30] and was approved by the Ethics Committee of the Universitat Autònoma de Barcelona (CEAAH 3451).

### Measures

#### Sociodemographics and anthropometrics

Participants reported information about age, gender, parental origin, and educational level. Parental educational level was based on the aggregated levels of education from ILOSTAT, based on the International Standard Classification of Education (ISCED), including the following levels: less than basic, basic, intermediate, advanced, and level not stated [31].

Height in cm and weight in kg were measured using a SECA portable stadiometer, model 214 (20–207 cm; accuracy range of 0.1 cm), and SECA portable scales, model 8777021094 (0–200 kg; accuracy range of 0.1 kg), respectively. Weight status was calculated based on z-BMI scores, using the World Health Organization growth reference criteria [32].

#### Experienced weight stigma

Experienced weight stigma was assessed based on the approach proposed by several multinational survey-based studies [26, 33, 34] by using the following three yes/no questions: (1) “Have you ever been teased because of your weight?”; (2) “Have you ever been treated unfairly because of your weight?”; (3) “Have you ever been discriminated against because of your weight?”. Experienced any weight stigma was considered if individuals responded “yes” to at least one of the items. In addition, those who reported having experienced weight stigma were asked to indicate the frequency of experiencing weight stigma on a scale from 1 (Once) to 3 (Multiple times). The Internal consistency coefficients obtained in our sample were Cronbach’s alpha α = 0.82 and McDonald’s omega ω = 0.82.

#### Sources and frequency of weight-based victimization

To better understand the magnitude of the problem, we adapted a measure for assessing stigmatizing situations from previous research [35] to explore multiple sources of stigma and their frequency, in the following way: “Have you ever been teased, harassed, treated unkindly or made to feel bad or uncomfortable because of your weight?”. The piece “or made to feel bad or uncomfortable” was added to capture the negative affective experience associated with weight stigma. Frequency was assessed by a 5-point Likert scale ranging from “Never” to “Always”, and covering different sources of stigma, including school/institute, family I live with, other family, friends, other kids of my age, healthcare providers, strangers, media, society in general, and others. The Internal consistency coefficients obtained in our sample were Cronbach’s alpha α = 0.87 and McDonald’s omega ω = 0.86.

#### Weight bias internalization

We used the Modified Weight Bias Internalization Scale (WBISM) [36] in its Spanish validation for adolescents [37]. The WBISM measures weight self-stigma across the body weight statuses (e.g., “I hate myself for my weight”). The Spanish validated version used has 10 items with responses rated on a 7-point Likert scale, ranging from 1 (strongly disagree) to 7 (strongly agree). The Spanish validation for adolescents of WBISM has shown a high internal consistency and showed a unidimensional structure with an adequate fit and adequate construct validity [37]. In our sample, WBISM has shown a high internal consistency (α = 0.94; ω = 0.94) and showed a unidimensional structure with adequate fit (CFA: GFI = 0.998; PNFI = 0.665; NFI = 0.997; SRMR = 0.035). The mean of the item responses serves as the participant’s score (range 1–7), with higher scores indicating higher internalized weight bias. High levels of internalized weight stigma were established as WBISM scores greater than or equal to 4 (midpoint). This criterion has been recently used both in epidemiological research [21] and as a criterion for selecting eligible participants with high levels of internalized weight stigma in the research on the efficacy of treatments to reduce internalized weight stigma [38].

### Data analysis

Statistical analyses were performed with STATA 17 software. The significance level was set at 0.05 and hypothesis tests were two-sided.

Sociodemographic and anthropometric characteristics, experienced weight stigma, and frequency of weight stigma sources were described using number (n) and percentage (%) or mean and standard deviation (SD) as appropriate. To characterize the distribution of WBI, percentiles of the WBISM scores were calculated for the total sample and stratified by gender and weight status.

To describe the prevalence of experienced weight stigma and high levels of WBI, the number (n) and percentage (%) with the corresponding 95% confidence interval (95% CI) were provided. Fisher’s exact test and multivariate logistical regression models were performed to determine their association with sociodemographic variables and weight status. In order to address concerns regarding potential cluster bias, we initially conducted a mixed-effects (multilevel) logistic regression analysis, with course and school as hierarchical levels for both dependent variables. Upon evaluating empty models and the intraclass correlation coefficient (ICC), our findings revealed minimal variability both between courses and schools, with ICC values consistently below the threshold of 0.05, threshold often regarded as a conventional indicator of the presence of clustering [39]. Furthermore, likelihood ratio tests comparing the multilevel models to standard binary logistic regression models showed no significant improvement in model fit (*p* > .05). Given the lack of meaningful variation between courses and schools, we opted for the more parsimonious approach of employing simple logistic regression models for our analyses. To assess the strength of association, adjusted odds ratios (AOR) with corresponding 95% CI were provided. The goodness-of-fit of the multivariate logistic regression model was tested using the Hosmer-Lemeshow test, and the R^2^ value was used to express the proportion of variance explained by the model.

## Results

The mean age of the participants was 14.4 years (SD = 1.2), with girls constituting 48.9% of the sample. Since there were only 3 individuals identified as non-binary, the analyses involving gender were confined to girls and boys. The characteristics of the sample are shown in Table 1.

**Table 1:** Sociodemographic and anthropometric characteristics of the sample (*n*=1016)

	Total	
	*n*	%
**Gender**		
Girls	497	48.92
Boys	516	50.79
Non-binary	3	0.30
**Parental origin**		
Spain	744	73.23
Europe	11	1.08
Latin America	56	5.51
North Africa	86	8.46
Mixed	86	8.46
Other	33	3.25
**Parental educational level**		
Less than basic	8	0.79
Basic	194	19.09
Intermediate	283	27.85
Advanced	319	31.40
Level not stated	212	20.87
**Age (years)**		
11–12	173	17.02
13–14	504	49.61
≥15	339	33.33
**Weight status**		
Underweight	14	1.38
Normal weight	726	71.46
Overweight	199	19.59
Obesity	77	7.48

### Prevalence of experienced weight stigma

Table 2 provides the crude prevalence of experienced weight stigma by gender, parental origin, parental educational level, age, and weight status, and also the prevalence of weight stigma by weight status stratified by gender. Overall, the prevalence of adolescents who reported experiencing some type of weight-related stigma was 43.2%. No significant association was found between experienced weight stigma and either parental origin or parental educational level. However, being a girl, being older, and having a higher weight status were significantly associated with having experienced weight stigma. Besides, despite weight stigma being reported across all weight statuses, including those with normal weight (35% for the whole sample), the prevalence of weight stigma was particularly higher among adolescents with obesity (82%).

**Table 2:** Prevalence of experienced weight stigma and high levels of WBI (WBISM ≥ 4) by sociodemographics, weight status, and by weight status stratified by gender (*n*=1016)

	Experienced weight stigma				WBISM ≥ 4			
	*n*	%	95% CI		*n*	%	95% CI	
**Total**	439	43.21	(40.25, 46.33)		197	19.39	(17.07, 21.94)	
**Gender**	***p*** **< .001**				***p*** **< .001**			
Girls	258	51.91	(47.51, 56.28)		149	29.98	(26.11, 34.16)	
Boys	179	34.69	(30.70, 38.91)		47	9.11	(6.91, 11.92)	
Non-binary	NA				NA			
**Parental origin**	*p* = .562				*p* = .364			
Europe	322	42.65	(39.16, 46.21)		141	18.68	(16.05, 21.76)	
Other	117	44.83	(38.89, 50.91)		56	21.46	(16.89, 26.86)	
**Parental educational level**	***p*** **= .460**				***p*** **= .870**			
Less than basic	4	50.00	(19.98, 80.02)		1	12.50	(1.72, 53.79)	
Basic	82	42.27	(35.50, 49.34)		42	21.65	(16.41, 28.01)	
Intermediate	135	47.70	(41.93, 53.53)		56	19.79	(15.55, 24.85)	
Advanced	130	40.75	(35.48, 46.24)		61	19.12	(15.17, 23.82)	
Level not stated	88	41.51	(35.06, 48.26)		37	17.45	(12.91, 23.17)	
**Age (years)**	***p*** **= .044**				***p*** **= .904**			
11–12	64	36.99	(30.12, 44.44)		34	19.65	(14.39, 26.26)	
13–14	212	42.06	(37.82, 46.43)		95	18.85	(15.67, 22.51)	
≥15	163	48. 08	(42.80, 53.41)		68	20.06	(16.13, 24.67)	
**Weight Status**	***p*** **< .001**				***p*** **< .001**			
Underweight	8	57.14	(31.60, 79.37)		4	28.57	(11.13, 56.09)	
Normal weight	257	35.40	(32.00, 38.95)		101	13.91	(11.58, 16.63)	
Overweight	111	55.78	(48.80, 62.53)		53	26.63	(20.95, 33.21)	
Obesity	63	81.82	(71.59, 88.93)		39	50.65	(39.62, 61.62)	
**Girls**								
**Weight Status**	***p*** **< .001**				***p*** **< .001**			
Underweight	1	50.00	(5.85, 94.15)		3	50.00	(5.85 94.15)	
Normal weight	171	45.84	(40.84, 50.94)		88	23.06	(19.05 27.62)	
Overweight	61	63.54	(53.47, 72.55)		26	42.71	(33.20 52.79)	
Obesity	26	96.15	(77.12, 99.46)		32	80.77	(61.24 91.78)	
**Boys**								
**Weight Status**	***p*** **< .001**				***p*** **< .001**			
Underweight	7	58.33	(30.71, 81.56)		4	24.00	(8.25, 55.26)	
Normal weight	86	24.43	(20.22, 29.20)		13	3.98	(2.37, 6.61)	
Overweight	49	48.04	(38.51, 57.71)		5	11.76	(6.79, 19.61)	
Obesity	37	74.00	(60.17, 84.28)		25	36.00	(23.97, 50.09)	

*WBISM* Modified Weight Bias Internalization Scale, *95% CI* 95% Confident Interval, *p* = Fisher’s exact test significance, *NA* not applicable for insufficient sample

**Fig. 2:**
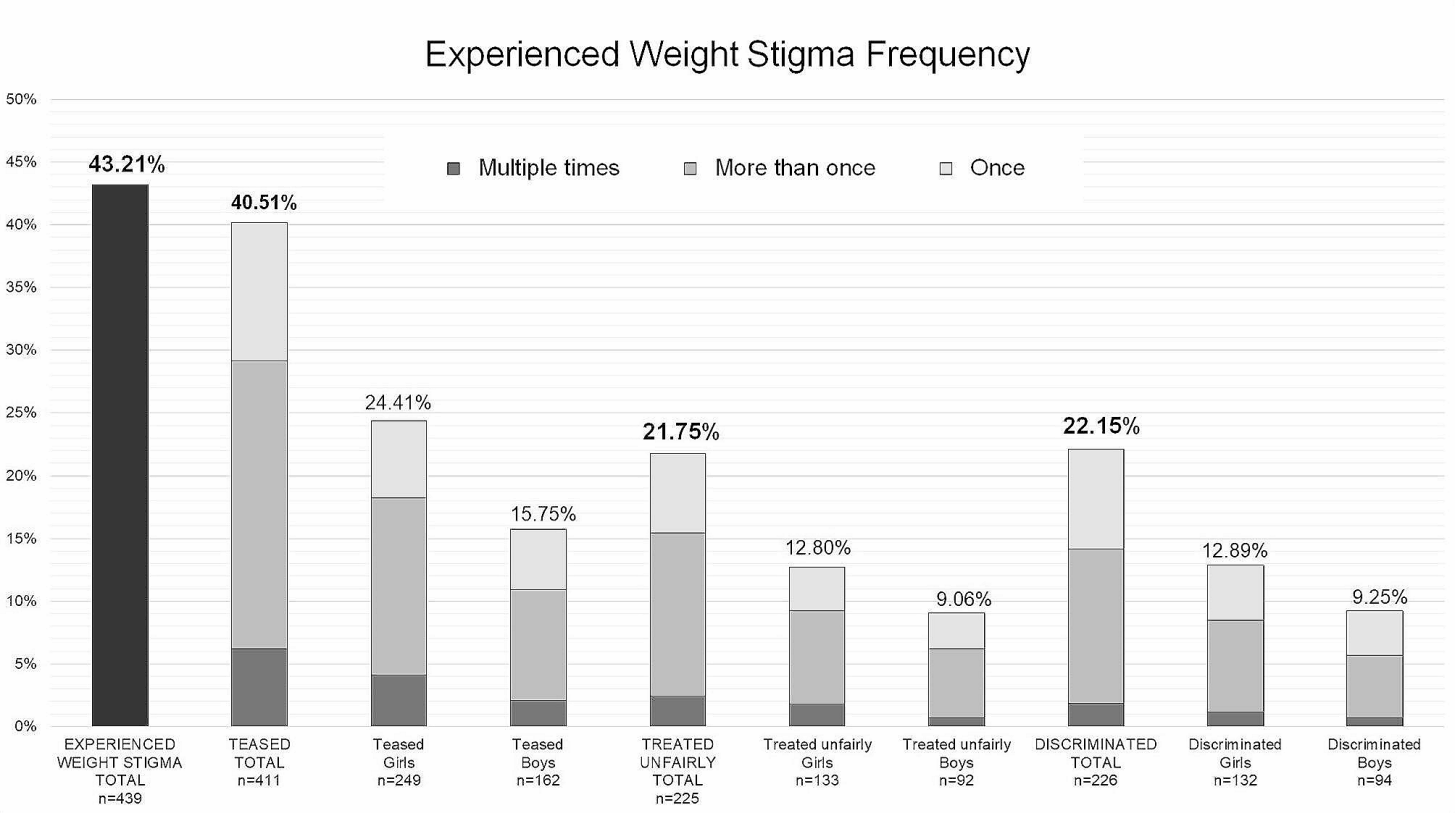
Frequency of different types of experienced weight stigma

The prevalence of experienced weight stigma was 96.2% for girls with obesity and 74% for boys with obesity, highlighting differences in prevalence when both gender and weight status were considered together. Figure 2 shows the frequencies of reported experiences of teasing, unfair treatment, or weight discrimination by gender.

Regarding the sources of weight stigma (Table 3), the most common sources for both girls and boys were the school/institute, other kids of the same age, followed by society for girls and friends for boys. It is worth noting that 12% of the entire sample indicated the family they lived with as a source of stigma, with this percentage rising to 20% among girls.

**Table 3:** Frequency of sources of weight stigma (*n*=1013)

	Girls		Boys		Total	
	*n*	%	*n*	%	*n*	%
**School / Institute**						
Never/Rarely	341	68.61	439	85.08	780	77.00
Sometimes/Often/Always	156	31.39	77	14.92	233	23.00
**Family I live with**						
Never/Rarely	397	79.88	491	95.15	888	87.66
Sometimes/Often/Always	100	20.12	25	4.84	125	12.34
**Other family**						
Never/Rarely	416	83.70	505	97.87	921	90.92
Sometimes/Often/Always	81	16.30	11	2.13	92	9.08
**Friends**						
Never/Rarely	435	87.53	460	89.15	895	88.35
Sometimes/Often/Always	62	12.47	56	10.85	118	11.65
**Other kids my age**						
Never/Rarely	332	66.80	431	83.53	763	75.32
Sometimes/Often/Always	165	33.20	85	16.47	250	24.68
**Healthcare providers**						
Never/Rarely	449	90.34	501	97.09	950	93.78
Sometimes/Often/Always	48	9.66	15	2.91	63	6.22
**Strangers**						
Never/Rarely	408	82.09	473	91.66	881	86.97
Sometimes/Often/Always	89	17.91	43	8.33	132	13.03
**Media**						
Never/Rarely	451	90.74	507	98.25	958	94.57
Sometimes/Often/Always	46	9.26	9	1.74	55	5.43
**Society in general**						
Never/Rarely	358	72.03	470	91.09	828	81.74
Sometimes/Often/Always	139	27.97	46	8.91	185	18.26
**Others**						
Never/Rarely	430	87.93	487	95.12	917	91.61
Sometimes/Often/Always	59	12.07	25	4.88	84	8.39

### Prevalence of internalized weight stigma

The overall mean score for WBISM in our sample was 2.5 (SD = 1.6). As shown in Table 2, the prevalence of high levels of WBI (WBISM ≥ 4) in our sample was 19.4% (95% CI: 17.1 to 21.9), with higher WBI observed among girls and adolescents with obesity. When both gender and weight status were considered together (Table 2), girls across all weight statuses reported a higher prevalence of WBI compared to boys. Specifically, only 4% of boys with normal weight reported high levels of WBI, while this percentage exceeded 20% in girls with normal weight and rose up to 80% in girls with obesity.

Given the observed disparities in high levels of WBISM across gender and weight status, we computed percentile ranks specific to each category, which are presented in Table 4. As indicated, the 75th percentile of the WBISM scores was p75 = 3.4 for the whole sample, but this increased to p75 = 4.3 in girls and p75 = 5.1 in adolescents with obesity.

**Table 4:** Percentiles of the WBISM scores, for the total sample and stratified by gender and weight status

	Total	Gender *n* = 1013		Weight status *n* = 1016			
Percentile		Girls	Boys	Underweight	Normal weight	Overweight	Obesity
10	1.0	1.0	1.0	1.0	1.0	1.1	1.7
20	1.1	1.3	1.1	1.0	1.1	1.3	2.1
25	1.2	1.5	1.1	1.1	1.1	1.5	2.4
30	1.3	1.6	1.1	1.6	1.2	1.7	2.7
40	1.6	1.9	1.3	1.8	1.4	2.1	3.2
50	1.9	2.7	1.6	2.2	1.6	2.7	4.0
60	2.4	3.2	1.8	2.7	2.0	3.2	4.5
70	3.0	3.9	2.2	2.7	2.5	3.8	4.7
75	3.4	4.3	2.4	4.3	2.8	4.1	5.1
80	3.9	4.6	2.8	4.7	3.2	4.7	5.6
90	5.1	5.7	3.9	4.8	4.4	5.7	6.2
95	5.9	6.2	5.0	6.1	5.3	6.3	6.8
99	6.7	6.8	6.1	6.1	6.3	6.9	7.0

*WBISM* Modified Weight Bias Internalization Scale

### Association of weight stigma and internalized weight stigma (AOR) with sociodemographic variables and weight status

As shown in Table 5, when assessing the AOR, gender, age, and weight status showed a statistically significant association with reported experiences of stigmatization. The adjusted odds were notably higher in girls (AOR = 2.6; 95% CI: 2.0 to 3.4) and older adolescents (AOR = 1.9; 95% CI: 1.3 to 3.0). When compared to normal weight adolescents, all other weight statuses showed higher adjusted odds, being 3.4 times higher (95% CI: 1.1 to 10.1) in adolescents with underweight and reaching 11.4 times higher odds (95% CI: 6.1 to 21.3) in those with obesity.

**Table 5:** Results of multivariate logistic regression models (AOR) for experienced weight stigma and high levels of WBI (WBISM ≥ 4) (*n*=1013)

	Experienced weight stigma		WBISM ≥ 4	
	AOR	(95% CI)	AOR	(95% CI)
**Gender**				
Boys	Ref.		Ref.	
Girls	**2.60**	**(1.97, 3.42)**	**6.63**	**(4.36, 10.07)**
**Age groups (years)**				
11–12	Ref.		Ref.	
13–14	1.43	(0.97, 2.11)	1.09	(0.67, 1.77)
≥15	**1.95**	**(1.28, 2.96)**	1.21	(0.71, 2.04)
**Parental educational level**				
Less than basic	Ref.		Ref.	
Basic	0.53	(0.12, 2.39)	1.31	(0.13, 12.78)
Intermediate	0.77	(0.17, 3.47)	1.45	(0.15, 14.09)
Advanced	0.58	(0.13, 2.61)	1.34	(0.14, 13.00)
Level not stated	0.69	(0.15, 3.13)	1.28	(0.13, 12.62)
**Parental origin**				
Europe	Ref.		Ref.	
Other	1.01	(0.74, 1.37)	1.11	(0.75, 1.64)
**Weight status**				
Normal	Ref.		Ref.	
Underweight	**3.37**	**(1.12, 10.12)**	**6.33**	**(1.76, 22.76)**
Overweight	**2.51**	**(1.80, 3.49)**	**2.66**	**(1.77, 4.00)**
Obesity	**11.39**	**(6.10, 21.26)**	**13.11**	**(7.28, 23.63)**

*WBISM* Modified Weight Bias Internalization Scale, *AOR* Adjusted Odds Ratio, *95% CI* 95% Confidence Interval, Ref.: reference category. In bold: *p* < 0.05

Regarding high levels of internalized weight stigma (WBIS ≥ 4), only gender and weight status showed a significant association. The adjusted odds ratio observed in girls was 6.6 times higher than in boys (95% CI: 4.4 to 10.1). Compared to normal weight adolescents, once again all other weight statuses showed higher adjusted odds. Specifically, the odds were 6.3 times higher (95% CI: 1.8 to 22.8) in adolescents with underweight, with the highest odds observed in adolescents with obesity, being 13 times higher than those of normal weight (95% CI: 7.3 to 23.6).

The Hosmer-Lemeshow chi^2^ goodness-of-fit statistic confirmed the adequacy of the model fit (*p* > .05). The proportion of variance explained (R^2^) by the models was 10.06% (*p* < .001) for experienced weight stigma and 16.43% (*p* < .001) for high levels of internalized weight stigma (WBIS ≥ 4).

## Discussion

This is the first study, to our knowledge, to provide prevalence data on a full spectrum of weight-related stigmatizing experiences and their internalization in a representative sample of adolescents from a large Spanish city, and their association with several sociodemographic variables and weight status.

Although the prevalence of obesity in youth has nearly quadrupled since 1990 [4], there is no indication of a decline in the stigmatization of individuals with higher body weights. On the contrary, obesity stigma and its internalization are becoming highly prevalent in our society, making youth, regardless of their body size, particularly vulnerable to emotional and physical health problems over time [13, 20]. Our study validated these prior findings underscoring the high prevalence of weight-related stigma experiences among adolescents, which exceeds 40% in the total sample and reached practically all girls with obesity. Previous studies using similar criteria have consistently demonstrated high prevalence rates of weight stigma experiences among community samples of adults. Specifically, one study reported a prevalence rate of 57% [21], while international comparisons across different countries involving adults engaged in weight loss programs reported stigma experience prevalence rates ranging from 56 to 61% [33]. Furthermore, high levels of WBI (almost 20%) were observed in the sample. High levels of WBI were observed in community samples of adults, reaching 24% [21]. A prevalence of 20.7% of high levels of WBI was recently found in another sample of Spanish adolescents [27], very similar to ours, supporting that high levels of WBI are around 20% in Spanish adolescents^1^.

In relation to the sources of stigma, our findings revealed that among adolescents who had experienced stigma, the most prevalent sources of stigma were other kids of their age and school, both approaching 25%. Notably for girls, society in general (approaching 30%) and family (about 20%) were reported as other primary sources of stigma. These findings are consistent with the association between societal pressure to conform to the thin ideal and increased weight bias in females [40]. Furthermore, our findings, both for girls and boys, corroborate those from a previous systematic review on the impact of stigma on youth mental health [41], which identified family members as one of the most frequent sources of stigma in youth together with peers and friends. Family-based weight stigma, particularly from mothers, is a widespread issue globally and is associated with various negative psychological outcomes [42]. Behaviors such as encouraging dieting and making critical comments about weight status by parents contribute to unhealthy eating behaviors and poor self-perception among youth [43, 44]. Moreover, experiences of family-based weight stigma increase the risk of eating disorders and emotional problems in both the short- and long-term [45–47]. Notably, mothers’ critical comments and dietary habits have a significant impact on weight stigma internalization in adolescents [48]. These findings underscore the importance of reducing weight stigma and emphasize the need for family members to distinguish between supportive, encouraging communication and potentially weight-stigmatizing discourse.

When looking at the prevalence of stigmatizing experiences by sample characteristics, in terms of gender differences, our results showed that adolescent girls were at a 2.6 times higher risk of having experienced stigma and a 6.6 times higher risk of having high levels of WBI compared to boys, which aligns with previous studies among adolescent samples [27, 49] and adult populations [21, 36]. In our society, thinner body types are often normalized as more attractive for women, placing societal pressure on them to conform to these ideals [50]. This pressure, when perceived by women, triggers an increased desire to meet these expectations, consequently making them more vulnerable to WBI [40].

Relative to weight status, it is concerning that about 35% of adolescents classified as having a normal weight reported experiences of weight-related discrimination, teasing, or bullying, in line with previous research with an adult sample [21]. Furthermore, our results suggested that individuals with lower and higher weight status were more vulnerable to experiencing weight stigma compared to individuals within the normal weight range. The association between higher BMI and an increased likelihood of experiencing weight stigma among young individuals (11.4 times higher risk in our study) is supported by a previous review of the literature [13]. Interestingly, adolescents with underweight from our sample also seemed to experience more weight-related discrimination compared to their normal-weight counterparts, a finding that aligns with recent research which indicates that individuals with underweight may be seen as unattractive or potential disease threats, leading to their stigmatization [51].

As regards WBI, a similar trend to that of experienced weight stigma was observed, supporting the existence of a “J-shaped” pattern of weight stigma internalization at the lowest and highest BMIs, although the highest risk of experiencing high levels of WBI occurred again in adolescents with obesity, with a risk 13.1 times higher than in adolescents with normal weight. Our findings align with previous research conducted on a sample of Spanish adolescents [27] and with a study conducted with a community sample of adults [21], which reported a 9.4 times higher risk of individuals with obesity having high WBI scores compared to those with normal weight.

In terms of age, our findings showed that weight stigma experiences and WBI tend to increase with age, being more prevalent among older adolescents compared to their younger counterparts. Previous studies conducted on adult samples [21, 40] indicated that weight stigma experiences were less prevalent among older individuals. Taken together, these results may indicate that weight stigma experiences and their internalization may reach a significant peak during late adolescence and young adulthood, and gradually decrease throughout the lifespan. Further studies are needed to investigate the impact of age on weight stigma experiences and WBI across the lifespan.

In line with previous research on adults [21], our study did not identify significant variations in weight stigma experiences and WBI based on educational level and parental origin. However, it is worth noting that another study did find higher weight bias (not WBI) among less educated individuals [40].

The present study has several strengths. The sample was drawn randomly through an attentive sampling method, unlike most studies in this field, which are conducted with convenience community samples. The carefully selected sample enhances the generalizability of our findings to the broader adolescent population in the region. Moreover, a 3-item scale was used to assess weight stigma experiences, the same procedure assessment followed by recent multinational studies on weight stigma, which facilitates result comparisons. A recent review on the impact of paediatric WBI highlighted the need for a consistent and reliable standard for measuring WBI among adolescents [20]. Among the studies identified in this review, the WBIS was the most common instrument used to measure WBI among children and adolescents, but often underwent adaptations to the wording of the items in order to accommodate younger respondents. Our study used the recently validated WBISM version for the Spanish adolescent population, one of the few validated versions of the WBIS for use with adolescents across weight categories. Our study employed an assessment of various sources of weight-based discrimination, including school, peers, family, media, healthcare, and society in general, and their frequency was included. By exploring the associations of these experiences across gender, age, parental origin, educational level, and weight status, the study provided a comprehensive understanding of the contextual factors that contribute to both weight stigma experiences and WBI among adolescents. Furthermore, the objective measurement of the weight and height of the participants ensures the accuracy of weight status data collection, while the majority of community studies on this topic rely on self-reported data.

Among the limitations of the study, it should be noted that, although the sample is representative of adolescents from a large city in Catalonia, Spain, caution is advised when generalizing these findings to other areas and regions. Further studies of this nature are necessary in Spain. The representativeness of the initial sample could be compromised by a significant number of families providing negative informed consent, declining to respond to the request for informed consent for their children, as well as other losses for various reasons, such as absenteeism. The reasons behind this high non-response rate can be attributed to two factors. Firstly, the impact of the COVID-19 pandemic played a role, as both the responsible professors for collecting informed consents and the families themselves experienced exhaustion and a reduced interest in activities unrelated to academics during the first year of “normalcy.” The argument is based on information provided directly by school administrators and some tutors when we asked about the possible reasons for the high number of negative informed consents. They explained to us that, following the COVID-19 pandemic, many families and teachers were fatigued by the changes demanded by the lockdown (for instance, implementation of digital learning systems, new assessment systems, etc.). Therefore, after returning to normalcy, any demand that involved deviating from the official academic program generated more rejection than usual from families and little motivation in some tutors, who were ultimately responsible for obtaining the informed consents from students. Secondly, the growing issue of weight stigma might have deterred participation among young individuals and their families. This explanation has been also provided by the PASOS report from *the Gasol Foundation*, which reported lower participation in PASOS 2022 compared to PASOS 2019 [52]. If this were the case, the prevalence data of stigma experiences and WBI may also be underestimated. Furthermore, in the current and previous studies [21, 38], a cut-off score of 4 was used to identify “elevated” WBI. However, there is no universally accepted cut-off point for WBISM, which could hinder the ability to compare findings across different studies [53]. Efforts should be made to identify the most suitable method for the classification of WBI. Although the measure of weight stigma experiences has been widely used in previous research and in multinational survey-based studies, its validity and reliability have not yet been examined, an issue that remains to be addressed in future studies. In addition, this study employed a cross-sectional design, which limits the ability to establish causal relationships between weight stigma experiences and demographic correlates. The limited variance explained by our models could be attributed to the complex and multifactorial nature of both experienced and internalized weight stigma. Our models only address some of their possible determinants, underscoring the importance of studying the potential influence of individual, contextual, as well as cultural and social determinants in future research. Finally, some origins and gender minorities might be underrepresented in the study sample. For instance, previous studies on adolescent samples found higher levels of WBI within non-binary gender individuals compared to boys [27, 54]. Unfortunately, due to the limited sample size for non-binary adolescents in the present study, it was not possible to calculate prevalence rates specifically for them. However, there is a growing need for further exploration of WBI across diverse sexual orientation and gender identity groups, particularly among young individuals.

Our study significantly contributes to the global call for increased research on weight stigma, particularly in diverse languages and cultures [55]. By deepening our understanding of the determinants of weight-related stigma and its internalization in culturally diverse populations, we can develop effective interventions and policies that promote inclusivity and mitigate the harmful effects of stigma. Our study provides valuable insights into the prevalence and sociodemographic determinants of weight stigma among a representative sample of adolescents from a big city in Spain. However, it also underscores the need for further research in this field in the Mediterranean region.

The high levels of weight bias experiences and WBI among our sample indicate the need to address these issues as potential risk factors in prevention and intervention strategies in community samples of adolescents of all body sizes. For this, it is important to consider that weight stigma is associated with two key factors: attributions of controllability to individuals with obesity and negative societal perceptions of obesity [56]. Consequently, individuals with overweight or obesity often face negative stereotypes, such as being perceived as lazy, gluttonous, lacking willpower and self-discipline, incompetent, unmotivated to improve health, non-compliant with medical treatment, and personally to blame for their higher body weight [57]. However, these beliefs are being challenged as obesity is increasingly being recognized as a complex chronic disease [4, 58–61]. Building upon these ideas, it’s noteworthy that organizations such as the World Obesity Federation [55] and UNICEF [62], have recently advocated for a change in the global obesity narrative to recognize and reduce weight stigma. Their recommendations include: reframing messages that oversimplify or attribute the causes of obesity solely to individual control; distinguishing between body size and obesity; using person-first language and non-stigmatizing imagery; showcasing visuals of the unhealthy food environments in which children grow up; avoiding language that blames parents or children; focusing on expanding people’s options rather than their choices; promoting weight-neutral health initiatives focused on health outcomes rather than weight; and raising awareness of weight stigma through professional and continuing education opportunities in education and healthcare contexts to improve equity for children and adolescents.

Based on the specific results of our study, strategies to address weight stigma should focus on vulnerable groups, such as girls and adolescents with obesity, and start at young ages. Regarding gender differences, specific preventive strategies should be tailored to address the thinness idealization among adolescent girls and enhance their resilience against societal pressures. In addition, stigmatizing experiences can take place in various settings. While our study revealed that the school setting is where the highest level of stigmatizing experiences occurs, comprehensive anti-bullying initiatives should extend beyond school and encompass multiple sources of weight stigma, including adolescents’ homes, clinical settings, and the media. These recommendations align with those from a growing group of international guidelines and statements aimed at ending weight stigma [15, 57, 63, 64] as well as with the more recent Spanish national strategic plan for the prevention of obesity [65].

## Data Availability

The datasets used and/or analysed during the current study are available from the corresponding author on reasonable request.

## Footnotes

1 This prevalence data is a personal communication from the authors of the cited study, even though it does not appear in the published paper.

## Abbreviations

AOR: Adjusted odds ratio
WBISM: Modified Weight Bias Internalization Scale
